# Muscle strength deficiency and mitochondrial dysfunction in a muscular dystrophy model of *Caenorhabditis elegans* and its functional response to drugs

**DOI:** 10.1242/dmm.036137

**Published:** 2018-12-04

**Authors:** Jennifer E. Hewitt, Amelia K. Pollard, Leila Lesanpezeshki, Colleen S. Deane, Christopher J. Gaffney, Timothy Etheridge, Nathaniel J. Szewczyk, Siva A. Vanapalli

**Affiliations:** 1 Department of Chemical Engineering, Texas Tech University, Lubbock, TX 79409, USA; 2 MRC/ARUK Centre for Musculoskeletal Ageing Research, Royal Derby Hospital, University of Nottingham & National Institute for Health Research Nottingham Biomedical Research Centre, Derby, UK; 3 Sport and Health Sciences, University of Exeter, St Luke's Campus, Exeter EX1 2LU, UK; 4 Lancaster Medical School, Furness College, Lancaster University, Lancaster LA1 4YG, UK

**Keywords:** Muscular dystrophy, *C. elegans*, Muscle strength, Prednisone, Melatonin

## Abstract

Muscle strength is a key clinical parameter used to monitor the progression of human muscular dystrophies, including Duchenne and Becker muscular dystrophies. Although *Caenorhabditis elegans* is an established genetic model for studying the mechanisms and treatments of muscular dystrophies, analogous strength-based measurements in this disease model are lacking. Here, we describe the first demonstration of the direct measurement of muscular strength in dystrophin-deficient *C. elegans* mutants using a micropillar-based force measurement system called NemaFlex*.* We show that *dys-1(eg33)* mutants, but not *dys-1(cx18)* mutants, are significantly weaker than their wild-type counterparts in early adulthood, cannot thrash in liquid at wild-type rates, display mitochondrial network fragmentation in the body wall muscles, and have an abnormally high baseline mitochondrial respiration. Furthermore, treatment with prednisone, the standard treatment for muscular dystrophy in humans, and melatonin both improve muscular strength, thrashing rate and mitochondrial network integrity in *dys-1(eg33)*, and prednisone treatment also returns baseline respiration to normal levels*.* Thus, our results demonstrate that the *dys-1(eg33)* strain is more clinically relevant than *dys-1(cx18)* for muscular dystrophy studies in *C. elegans*. This finding, in combination with the novel NemaFlex platform, can be used as an efficient workflow for identifying candidate compounds that can improve strength in the *C. elegans* muscular dystrophy model. Our study also lays the foundation for further probing of the mechanism of muscle function loss in dystrophin-deficient *C. elegans*, leading to knowledge translatable to human muscular dystrophy.

This article has an associated First Person interview with the first author of the paper.

## Abstract (precis)

**Editor's choice:** Dystrophin-deficient *Caenorhabditis* *elegans* have measurably weak muscle strength and mitochondrial dysfunction, and they respond to drug treatments standard in treating human Duchenne muscular dystrophy.

## INTRODUCTION

Duchenne muscular dystrophy (DMD) and Becker muscular dystrophy (BMD) are muscular wasting disorders that affect both skeletal and cardiac muscle and result from mutations in the dystrophin gene (Le Rumeur, 2015). Dystrophin is a protein encoded by the longest human gene, which is over 2.3 million base pairs long and has complex interactions with muscle contraction and muscle cell membrane stability (Den Dunnen et al., 1989; Blake et al., 2002). DMD results from null mutations in the gene, while BMD, a less severe form of the disorder, is typically caused by a mutation resulting in a partially functional dystrophin protein (Le Rumeur, 2015). The prevalence of these diseases is more than 1 in 4000 male births, expressing as an X-linked disorder. Prognosis is poor (Moser, 1984), and the only standard approved treatment in the USA for symptoms of DMD is the corticosteroid prednisone, which typically has the effect of extending ambulation by a couple of years (DeSilva et al., 1987). Although the increase in ambulatory period is a favorable outcome of treatment, chronic prednisone use typically results in a cushingoid appearance and other unfavorable side effects (Mendell et al., 1989; Malik et al., 2012). Thus, the prognosis and options for DMD/BMD treatment are rather limited.

To monitor progression of the disease or to test for efficacy of treatments, various diagnostic tools have been studied to monitor the deterioration of muscle in DMD patients. One diagnostic tool used is an electronic strain gauge that measures isometric muscle strength; this tool can discern DMD patients from the control in all muscle groups tested, with the most drastic differences occurring in the knee extensors, where DMD patients have less than a tenth of the strength of the control group (Brussock et al., 1992). Quantitative muscle testing (QMT), a method that is more sensitive to small changes in muscle strength, is also being implemented in young patients with DMD to monitor muscle strength across age. QMT is able to detect isometric and isokinetic losses in strength before the end of the first decade of life (Lerario et al., 2012). These are just two examples of a larger research effort to obtain more reliable measures of muscle strength, as muscle strength is regarded as a key clinical parameter of interest in tracking DMD disease progression. Over the past half century, research efforts surrounding muscular dystrophy have grown significantly, but we still have much to learn about this debilitating disease.

Although there have been extensive research efforts to better understand the mechanisms of and treatments for muscular dystrophy in vertebrate model organisms such as rodents and canines, these systems are limited in their throughput, can be cost prohibitive and also have some ethical issues (McGreevy et al., 2015). This has led researchers to utilize *Caenorhabditis* *elegans* to study muscular dystrophy over the past couple of decades (Ségalat, 2006; Chamberlain and Benian, 2000). *C. elegans* is a premier model organism for studying a number of biological processes and human diseases, with an estimated 40% of human disease genes having an ortholog in *C. elegans* (Culetto and Sattelle, 2000). The ability to translate results from *C. elegans* to humans comes, in part, from conserved major biological pathways between the two organisms and a fully sequenced nematode genome (The C. elegans Sequencing Consortium, 1998). *C. elegans* also has musculature strikingly similar to that of humans, with the presence of dense bodies (analogous to z-disks) and m-lines (Gieseler et al., 2016). A number of muscle proteins in *C. elegans* resemble human proteins in their function, making *C. elegans* an excellent model for studying muscle ailments such as sarcopenia or muscular dystrophy (Ségalat, 2002). In addition to these factors, *C. elegans* also has a short lifespan of only 3 weeks, produces a new generation every 3.5 days and is low maintenance, with cultures grown on agar medium and an *Escherichia* *coli* diet.

Several mutant strains of *C. elegans* have been generated for investigating the mechanistic details of and pharmacological treatments for dystrophin deficiency. About two decades ago, Bessou et al. reported a gene in *C. elegans* that they called *dys-1* (Bessou et al., 1998). *dys-1* encodes a protein resembling the human dystrophin protein not properly transcribed in DMD and BMD. These *C. elegans dys-1* mutants are hyperactive, have exaggerated head bending, hypercontract their bodies during backwards movements and are hypersensitive to the acetylcholinesterase inhibitor aldicarb. However, the animals do not show visible defects in their musculature, which the authors attribute to the short timescale of the nematode's life (Bessou et al., 1998). To address the need for a health measure related directly to the musculature in *dys-1* mutants, Gieseler et al. generated a sensitized *dys-1* mutant containing an additional mutation in the *hlh-1* gene, which is a homolog for the mammalian *MyoD* (also known as *Myod1*) gene (Gieseler et al., 2000). The presence of the *hlh-1* mutation in a *dys-1* mutant background results in significant muscle cell degeneration that is not present in mutants with either the *hlh-1* or *dys-1* mutation alone (Gieseler et al., 2000). This type of double mutation was modeled after a similar *MyoD* mutation studied in conjunction with the *mdx* mouse model, which was generated, in part, to create a system that recapitulated the pathophysiology of DMD in humans (Megeney et al., 1996). Using this *dys-1; hlh-1* model of muscular dystrophy, pharmacological compounds like prednisone and serotonin have been shown to be effective in reducing muscle cell degeneration. These two treatments came as hits out of large-scale screens, from which hundreds of other compounds were deemed ineffective (Gaud et al., 2004; Carre-Pierrat et al., 2006).

Although these studies have helped to establish *C. elegans* as a model organism for muscular dystrophy and pharmacological treatments for the disease, two main criticisms arise. First, it is unknown whether results from the *dys-1; hlh-1* double mutant models can be translated to muscular dystrophy in humans, especially given that the mechanism of these enhanced muscular degeneration effects in *C. elegans* is not fully understood. Second, although several assays for assessing health of *dys-1* mutants have been proposed, most fail to directly score animals for muscle function and instead look at indirect physiological parameters, such a locomotion speed, or subcellular markers, such as muscle cell damage. Beron et al. scored the percentage of worms that can travel a set distance in a certain amount of time when placed in a 3D burrowing environment (Beron et al., 2015). Animals were stimulated by chemotaxis to burrow down the length of a plastic pipette filled with agar, and *dys-1(cx18)* and *dys-1(eg33)* were both highly deficient in burrowing ability compared with the wild-type control. This work indicates that the *dys-1* mutants might be unable to burrow correctly due to defects in muscular strength.

Although these assays are undoubtedly valuable, the ability to directly evaluate muscle function would offer a more meaningful dimension for assessing the health of dystrophin mutants under treatments, given that strength is a clinical measure used to assess progression of DMD in humans. Previously our group established a novel technique and workflow for reliably measuring the muscle strength of *C. elegans*, independent of their behavior. This platform, NemaFlex, consists of a microfluidic device containing deformable pillars that the worm deflects as it crawls in the chamber. Nematode strength is scored from the maximal pillar deflections via a sophisticated image-processing software (Rahman et al., 2018). To establish strength as a phenotype of interest for assessing health in *dys-1* mutants, we used NemaFlex for studying two different *dys-1* strains, *dys-1(cx18)* and *dys-1(eg33)*, alongside the wild-type animal. We show that our platform can detect pharmacologically induced improvements by assessing the effects that melatonin and prednisone, compounds known to improve muscle health, have on the muscular strength of the same animals. We also evaluated whether the thrashing data and mitochondrial integrity for control and treatment groups agreed with the strength data. Finally, we show that mitochondrial network integrity and mitochondrial function are impaired in *dys-1(eg33)*, and treatment with prednisone repairs these defects. This work addresses the current gap in the ability to obtain strength measures in DMD model mutants, which will ultimately lead to a better understanding of muscular dystrophy. Additionally, our results indicate that *dys-1(eg33)* has a more pronounced and clinically relevant phenotype than what has been reported previously for *dys-1* mutants. We can detect our clinically relevant phenotype in the absence of the *hlh-1* sensitizing mutation, which better establishes *C. elegans dys-1* mutants as a useful model for studying muscular dystrophy.

## RESULTS

### *dys-1(eg33)*, but not *dys-1(cx18)*, worms are weaker than wild type

**Fig. 1.**
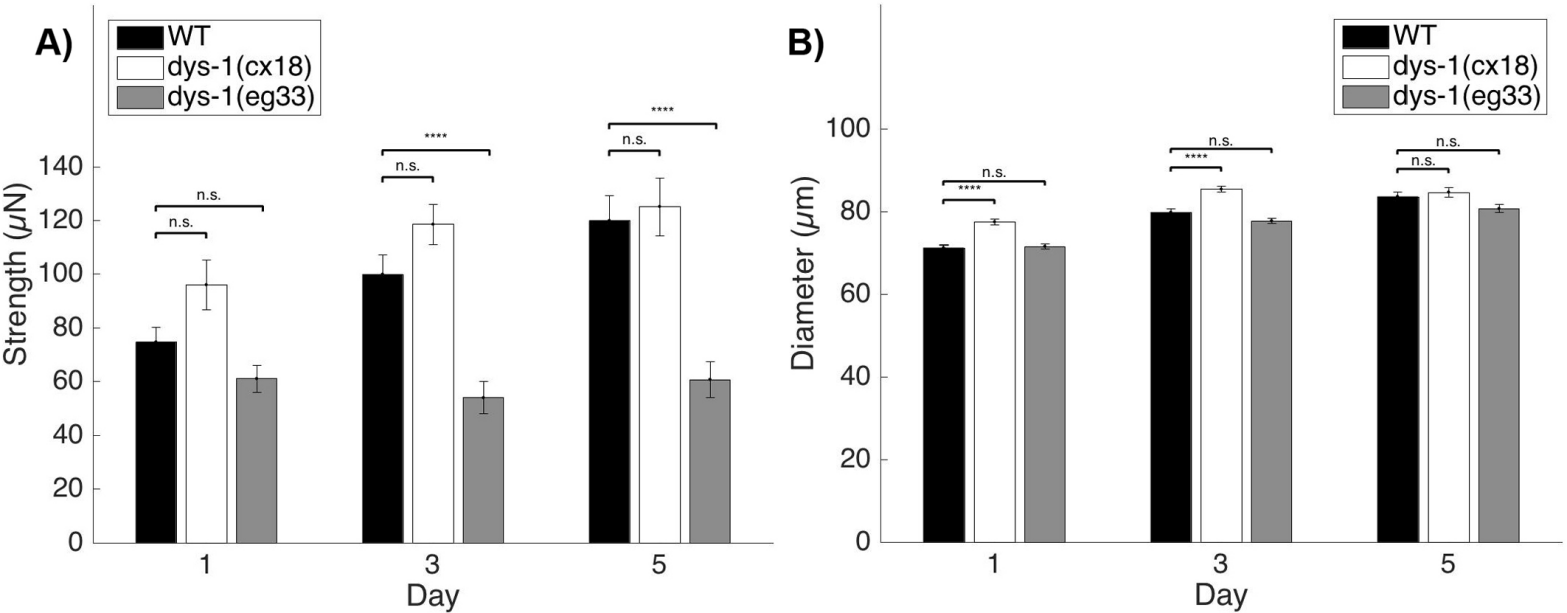
**Strength measurements of muscular dystrophy model mutants.** (A) Baseline strength of the three different strains taken at three different time points. Measurements began in early adulthood once animals had been transferred to the microfluidic devices. Error bars represent s.e.m. *dys-1(eg33)*, but not *dys-1(cx18)*, is detectably weaker than the wild-type (WT) animal. This effect of dystrophin loss on animal strength is detected beginning on Day 3. (B) The differences in animal strength are not attributable to their diameters, as *dys-1(eg33)* animals are weaker, but not thinner, than the WT animals. [*N* for Day 1, 3, 5: WT, *N*=27, 23, 22; *dys-1(cx18)*, *N*=29, 24, 22; *dys-1(eg33)*, *N*=28, 21, 18. Single replicate analyzed with a two-sample *t*-test.] ****P<0.0001; n.s., nonsignificant.

Although both *dys-1* mutants have previously been shown to have declined locomotory capability and decreased lifespan compared with the wild-type animal (Oh and Kim, 2013), direct measures of muscle functionality in clinically relevant models do not exist. We addressed this limitation by utilizing our microfluidic platform called NemaFlex that enables measurement of the muscular strength of *C. elegans* (Rahman et al., 2018). Using two previously studied dystrophin-deficient mutants, *dys-1(eg33)* and *dys-1(cx18)*, we investigated whether these animals were weaker than the wild-type animal (N2). The alleles *eg33* and *cx18* are nonsense mutations predicted to encode truncated forms of DYS-1 at amino acid (AA) 3287 and AA 2721, respectively (Oh and Kim, 2013). Animal strength of wild type, *dys-1(cx18)* and *dys-1(eg33)* was measured on Days 1, 3 and 5 of adulthood (Fig. 1A). Although neither mutant strength value was significantly different from that of wild type on the first day of adulthood, *dys-1(eg33)* animal strength essentially plateaued, whereas wild type and *dys-1(cx18)* continued to grow stronger at the later time points, which was potentially partially attributable to the increase in animal diameter in early adulthood. This led to *dys-1(eg33)* being significantly weaker than the wild-type control on Days 3 and 5, thus establishing the *dys-1(eg33)* strain as a model exhibiting muscular weakness with age, which is similar to the phenotype displayed in muscular dystrophy. It is important to note that animal diameter, but not length, strongly affects the muscle strength of *C. elegans*, as we previously reported that strength tends to increase with body diameter (Rahman et al., 2018). Therefore, we checked whether muscle strength deficiencies in *dys-1(eg33)* were attributable to differences in their diameters compared with wild-type animals (Fig. 1B). At no time point are *dys-1(eg33)* animals significantly thinner than wild type, thus indicating that their strength defect is not a size-based effect, and that we are truly measuring strength deficiencies resulting from defects in muscle function.

### All treatments improve *dys-1(eg33)* strength, some to wild-type levels

Because NemaFlex can detect muscular weakness in *dys-1(eg33)*, a meaningful next step is to test whether compounds known to improve muscle health can also improve muscle strength in muscular dystrophy models. Melatonin and prednisone were selected for validation of NemaFlex as a platform for screening compounds for treatment of dystrophin deficiency in *C. elegans*. Melatonin is thought to be potentially useful in treating muscle degradation with age (Coto-Montes et al., 2016) and has also been used to treat muscular dystrophy patients (Chahbouni et al., 2010). Prednisone is the standard treatment for muscular dystrophy patients (Malik et al., 2012) and has also been shown to decrease the number of abnormal muscle cells in the *dys-1; hlh-1* double mutant strain of *C. elegans* (Gaud et al., 2004). The mechanism behind prednisone's improvement in muscle function is still up for debate, but the efficacy of prednisone previously shown in *C. elegans* provides evidence that corticosteroids treat the muscle in ways other than reducing inflammation, given that *C. elegans* does not have an inflammatory pathway (Gaud et al., 2004).

**Fig. 2.**
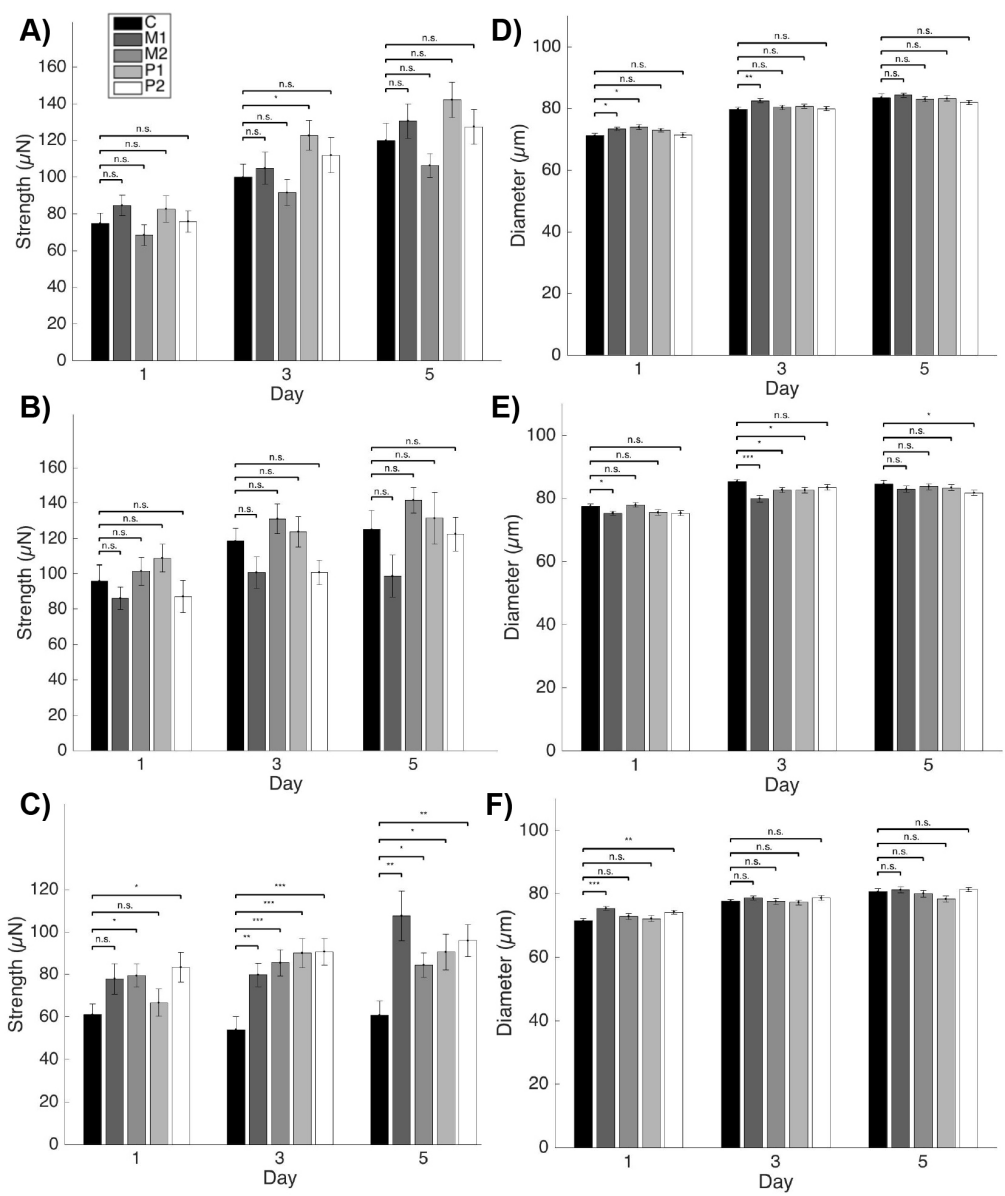
**Effect of pharmacological interventions on nematode strength.** (A-F) The strengths for three different strains, each with a control and four treatments, are shown. Each strain was treated with melatonin and prednisone during development alone (M1, P1) or during development and adulthood (M2, P2); ‘C’ designates the control animals, which received no treatment. With the exception of wild-type animals undergoing development-only prednisone treatment, the wild type (A) and *dys-1(cx18)* (B) have no changes in strength in response to treatment. In contrast, all *dys-1(eg33)* animals improve their strength under any of the four treatments beginning on Day 3 (C). Worm diameters do not fluctuate much for wild type (D), *dys-1(cx18)* (E) or *dys-1(eg33)* (F) under the various treatments. In the case of *dys-1(eg33)*, the diameter is not influenced at all by any treatments on Days 3 and 5, the time points at which strength improves drastically under treatment. These data indicate that strength improvements are not due to changes in animal size. Error bars represent s.e.m. [*N* for Day 1, 3, 5. WT: *M1*, *N*=27, 26, 25; *M2*, *N*=26, 25, 26; *P1*, *N*=26, 24, 23; *P2*, *N*=27, 24, 22. *dys-1(cx18)*: *M1*, *N*=29, 27, 25; *M2*, *N*=30, 24, 24; *P1*, *N*=28, 28, 21; *P2*, *N*=28, 28, 26. *dys-1(eg33)*: *M1*, *N*=27, 25, 23; *M2*, *N*=29, 25, 25; *P1*, *N*=29, 26, 23; *P2*, *N*=27, 25, 26. Single replicate analyzed with a two-sample *t*-test.] *P<0.05, **P<0.01, ***P<0.001; n.s., nonsignificant.

In general, we find that wild-type and *dys-1(cx18)* animals treated during development and continuing through adulthood were not significantly different from their control counterparts at all three time points (Fig. 2A,B). In contrast, beginning on Day 3 of adulthood, when *dys-1(eg33)* animals are significantly weaker than wild type, all four treatments improve muscular strength compared with the untreated *dys-1(eg33)* animals (Fig. 2C). Moreover, it is important to note that worm diameters are minimally affected under treatments for wild type (Fig. 2D), *dys-1(cx18)* (Fig. 2E) and *dys-1(eg33)* (Fig. 2F). Of particular importance is that on Days 3 and 5, when *dys-1(eg33)* has significant improvements in muscle strength, there are no changes in worm diameter under any treatment condition. Thus, improvements in animal strength are not due to changes in animal size, but rather due to improvements in muscle function. Under some treatments, differences between the wild-type control and treated *dys-1(eg33)* are indiscernible. Several treatments improve animal strength by over 50% and get within 10% of the wild-type control strength value. As anticipated, these treatments improve muscle functionality in the muscular dystrophy model in a manner that can be detected by NemaFlex. This establishes our technology as a useful platform for future studies screening novel compounds on *dys-1(eg33)* to select potential therapies for muscular dystrophy. Because *dys-1(eg33)* is showing such a distinct phenotype from *dys-1(cx18)*, which has been studied more thoroughly, we were interested in investigating the difference between these two strains and why *dys-1(eg33)* seems to have more clinical relevancy.

### Functional defects are apparent in swimming-based movement assays

**Fig. 3.**
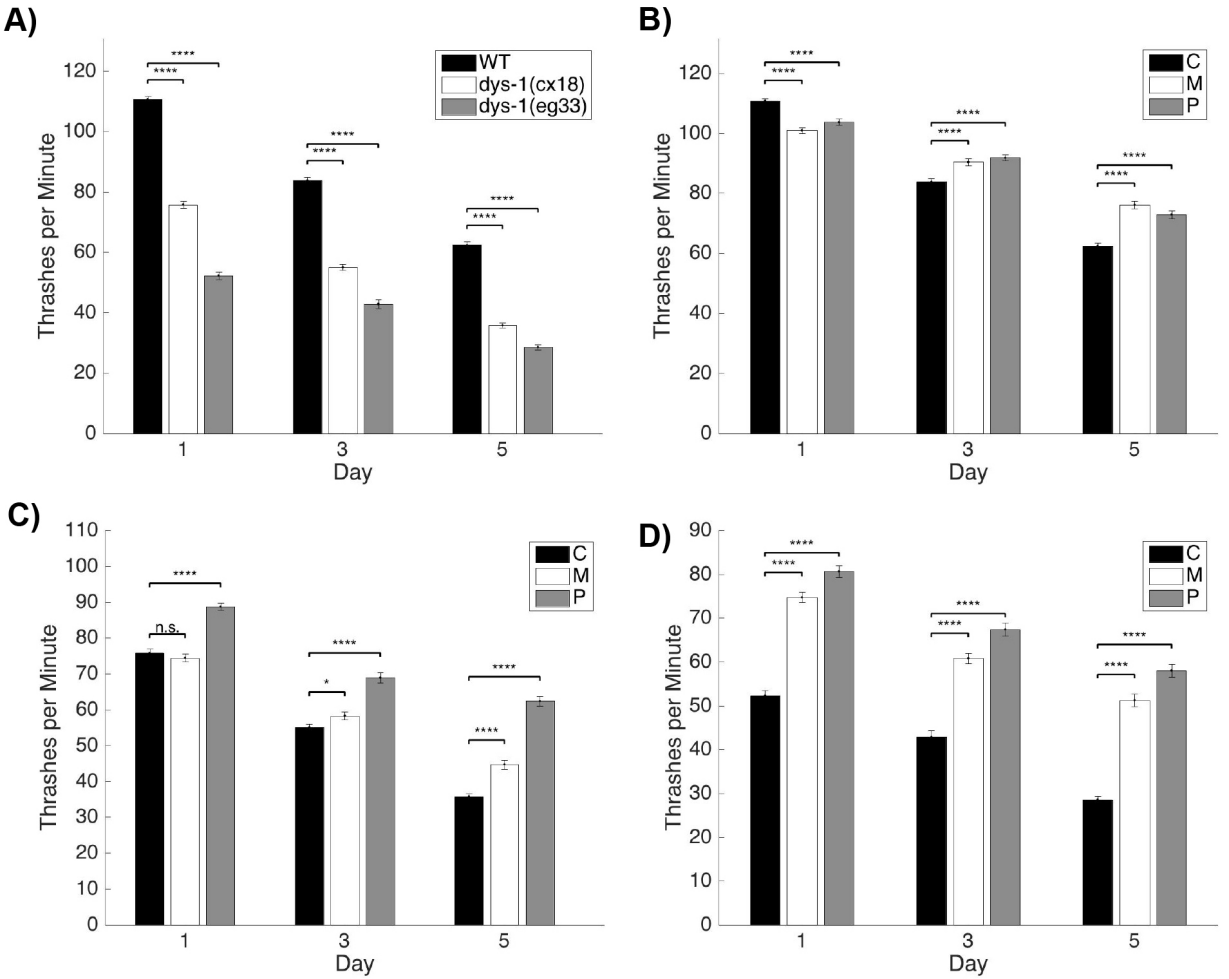
**Swimming dystrophin mutants have lower thrashing rates than wild type, and both *dys-1(cx18)* and *dys-1(eg33)* respond positively to treatments.** (A) Both *dys-1(cx18)* and *dys-1(eg33)* have lower thrashing rates than wild type (WT) across all ages. (B) WT, (C) *dys-1(cx18)* and (D) *dys-1(eg33)* have varying responses to drug treatments. The most prominent response is that of *dys-1(eg33)*, which improves its thrashing rate drastically under both treatments at all time points. C, control; M, melatonin across life (similar to previous M2 condition); P, prednisone across life (similar to previous P2 condition). Error bars represent s.e.m. For all strains and treatments at each time point, *N*=10, with five replicates for each worm, with three independent biological replicates for a total of 150 data points per bar; results were analyzed with a two-sample *t*-test. *P<0.05, ****P<0.0001; n.s., nonsignificant.

A standard assay for detecting locomotion defects is to record a worm's thrashing frequency when placed in a liquid environment, and this assay has been used previously to look at dystrophin-deficient worms, although not in both the *dys-1* strains we used in this study (Hueston and Suprenant, 2009). We were curious to compare the outputs of an indirect measure of muscle function, thrashing, with our more direct measure, the strength measurement. Interestingly, although the muscle strength of *dys-1(cx18)* was not significantly less than that of the wild-type animal, its thrashing rate was significantly less than that of wild type. *dys-1(eg33)* also showed a thrashing rate lower than that of both wild type and *dys-1(cx18)*, consistent with its lower strength (Fig. 3A). When all strains were treated with life-long melatonin or prednisone, there were some noticeable changes in the thrashing rate, although not the same as the changes in muscle strength in all cases. Wild-type animals had varying responses to the drugs, with the drugs not having a consistent effect on the worms across the time points studied (Fig. 3B,C). However, both treatments give a minor improvement in *dys-1(cx18)* (Fig. 3C), and they both offer a significant improvement in thrashing rate at all time points in *dys-1(eg33)* (Fig. 3D). This result matches well with the strength data, where all drug treatments improve muscle strength in *dys-1(eg33)*. The thrashing assay thus helps to further implement *dys-1(eg33)* as a more clinically relevant model, where measures from two unique modes of locomotion show improvement when animals are treated with compounds known to improve muscle health, particularly in patients with muscular dystrophy.

### *dys-1(eg33)* mutants have a more severe phenotype than *dys-1(cx18)*

**Fig. 4.**
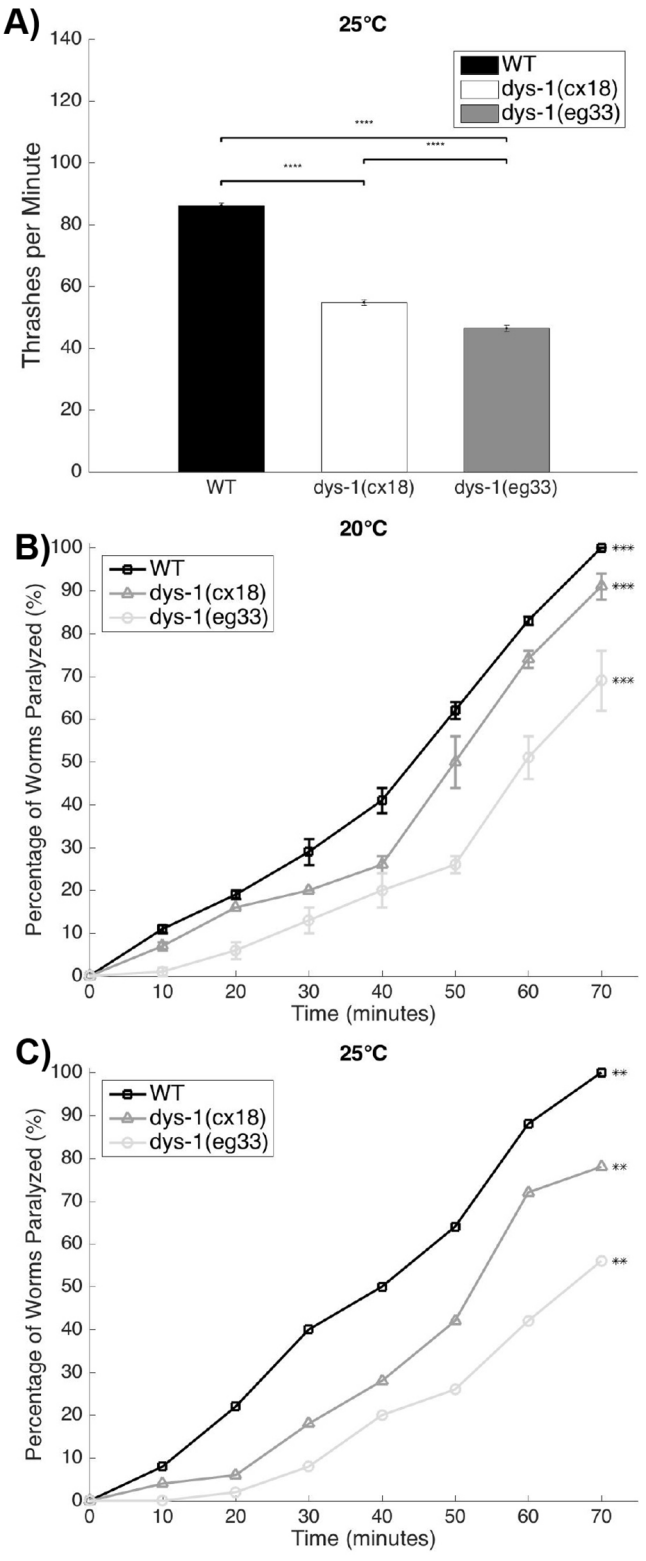
***dys-1(cx18)* shows a temperature-sensitive phenotype,** **and *dys-1(cx18)* and *dys-1(eg33)* are levamisole resistant.** (A) Day 1 adult *dys-1(cx18)* animals have lower thrashing rates when cultured at 25°C than at 20°C, whereas *dys-1(eg33)* is not affected by higher culture temperatures. Thus, *dys-1(cx18)* appears to be temperature sensitive. For all strains and treatments at each time point, *N*=10 with five replicates for each worm, with three independent biological replicates for a total of 150 data points per bar. Significances were analyzed using a two-way ANOVA with Tukey's multiple comparison test. (B,C) *dys-1(cx18)* has a mild levamisole resistance compared with wild type (WT), while *dys-1(eg33)* has a high resistance, both at 20°C and 25°C. At 20°C, *n*=50 for two independent biological replicates (total *n*=100 per strain); at 25°C, *n*=50 per strain. **P<0.01, ***P<0.001 and ****P<0.0001, for response to levamisole versus other strains tested. Two-way repeated measures ANOVA was used for statistical analysis.

Given that strength and thrashing ability are not compromised to the same extent in *dys-1(cx18)* as in *dys-1(eg33)*, we wanted to further investigate the differences between the two strains. Upon reviewing the published literature on *dys-1(cx18)*, we found that Hueston and Suprenant (2009) had previously observed worse locomotion at 25°C than at 20°C, which would be consistent with *cx18* being a temperature-sensitive allele. We confirmed that *dys-1(cx18)*, but not *dys-1(eg33)*, displays temperature sensitivity in the extent of thrashing ability (Fig. 4A). We next examined whether differences between *dys-1(cx18)* and *dys-1(eg33)* extended to differences in excitation-contraction coupling. Both *dys-1(cx18)* and *dys-1(eg33)* display resistance to levamisole-induced paralysis, indicative of defects in postsynaptic excitation-contraction coupling, with *dys-1(eg33)* displaying more pronounced levamisole resistance (Fig. 4B). Similar to the thrashing ability, *dys-1(cx18)* displayed temperature sensitivity to the effects of levamisole (Fig. 4C). These results confirm the past observation that *dys-1(cx18)* is a temperature-sensitive allele of *dys-1* and confirm that muscle responsiveness to a depolarizing signal is more compromised in *dys-1(eg33)*.

### Dystrophin mutants display normal sarcomere structure

**Fig. 5.**
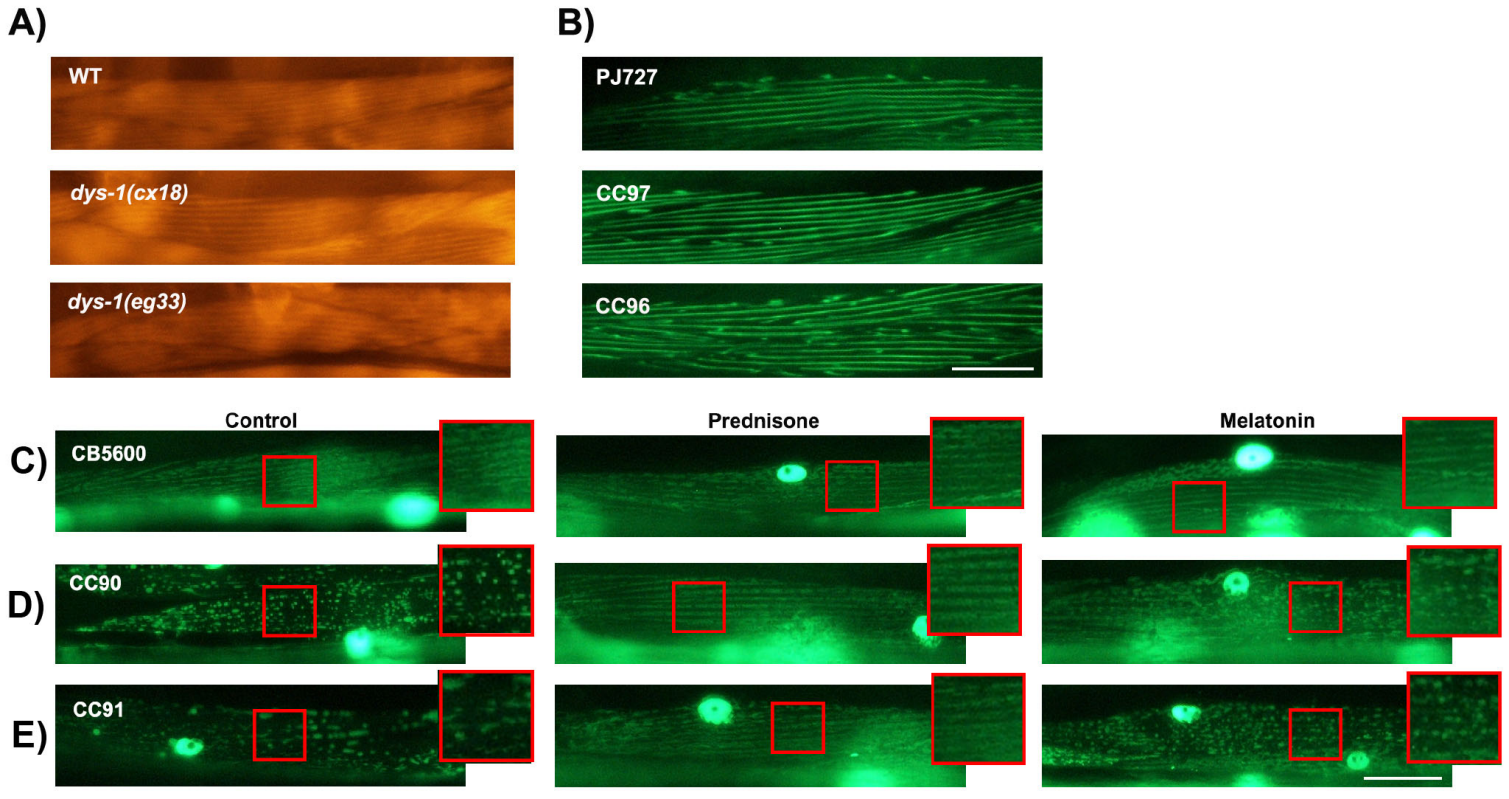
**There are no differences in sarcomere structure between *dys-1* and wild-type worms; however, mitochondrial network defects are apparent, and pharmacological intervention prevents degradation from occurring.** (A) Representative images of wild-type (WT), *dys-1(cx18)* and *dys-1(eg33)* worms stained with phalloidin on Day 1 of adulthood. (B) Representative images of PJ727, CC97 [*dys-1(cx18)*] and CC96 [*dys-1(eg33)*] worms on Day 3 of adulthood. Sarcomere defects are not apparent in either *dys-1* mutant. Scale bar: 25 μm. (C) CB5600 (WT with GFP-tagged mitochondria) animals have a tubular mitochondrial network appearance, which is also maintained in animals treated with prednisone and melatonin. (D) CC90 animals [GFP-tagged mitochondria in *dys-1(cx18)*] exhibit minor fragmentation in the mitochondrial network, which is remedied by prednisone but not melatonin. (E) CC91 animals [GFP-tagged mitochondria in *dys-1(eg33)*] have noticeably fragmented mitochondrial networks. Animals treated with prednisone do not display this phenotype and instead have relatively WT-like appearance in the mitochondrial network. Animals treated with melatonin have slightly improved mitochondrial network integrity but are not improved to WT levels. Scale bar: 25 μm; the enlarged regions are an additional 1.7× magnification.

We visualized the sarcomere structure of *dys-1(cx18)* and *dys-1(eg33)* worms to determine whether defects in muscle structure account for the reduced strength and motility in the *dys-1* worms. Similar to previous studies (Gieseler et al., 2000), we also detect no major differences in sarcomere structure in the *dys-1(eg33)* and *dys-1(cx18)* compared with wild-type worms, by either phalloidin staining (Fig. 5A) or visualization of myosin-tagged GFP (Fig. 5B). These findings suggest that the reductions in strength are not attributed to changes in muscle architecture in the *dys-1* strains and are perhaps a result of different mechanism(s).

### Mitochondrial fragmentation is a phenotype of dystrophin mutants

To determine the possible underlying mechanisms behind the loss of muscle strength in dystrophin mutants, we looked at the integrity of the mitochondrial network of *dys-1(cx18)* and *dys-1(eg33)* animals that had been crossed with the CB5600 strain, which expresses GFP in the mitochondria and nuclei of the body wall muscles. Recently, Scholtes et al. (2018) reported mitochondrial fragmentation as a phenotype of their sensitized muscular dystrophy strain, *dys-1; hlh-1*. Here, we report that mitochondrial network integrity is also compromised in *dys-1(cx18)* and *dys-1(eg33)* compared with wild-type animals of the same age, with the defect in *dys-1(eg33)* being more severe (Fig. 5C-E). Both prednisone and melatonin improve the mitochondrial integrity of *dys-1(eg33)* animals. This offers a potential mechanistic explanation for why muscle function appears to be more severely affected in *dys-1(eg33)* than in *dys-1(cx18)*, as well as further evidence that prednisone and melatonin are directly improving muscle health in *dys-1(eg33)*.

### Mitochondrial function is affected in *dys-1(eg33)* mutants

**Fig. 6.**
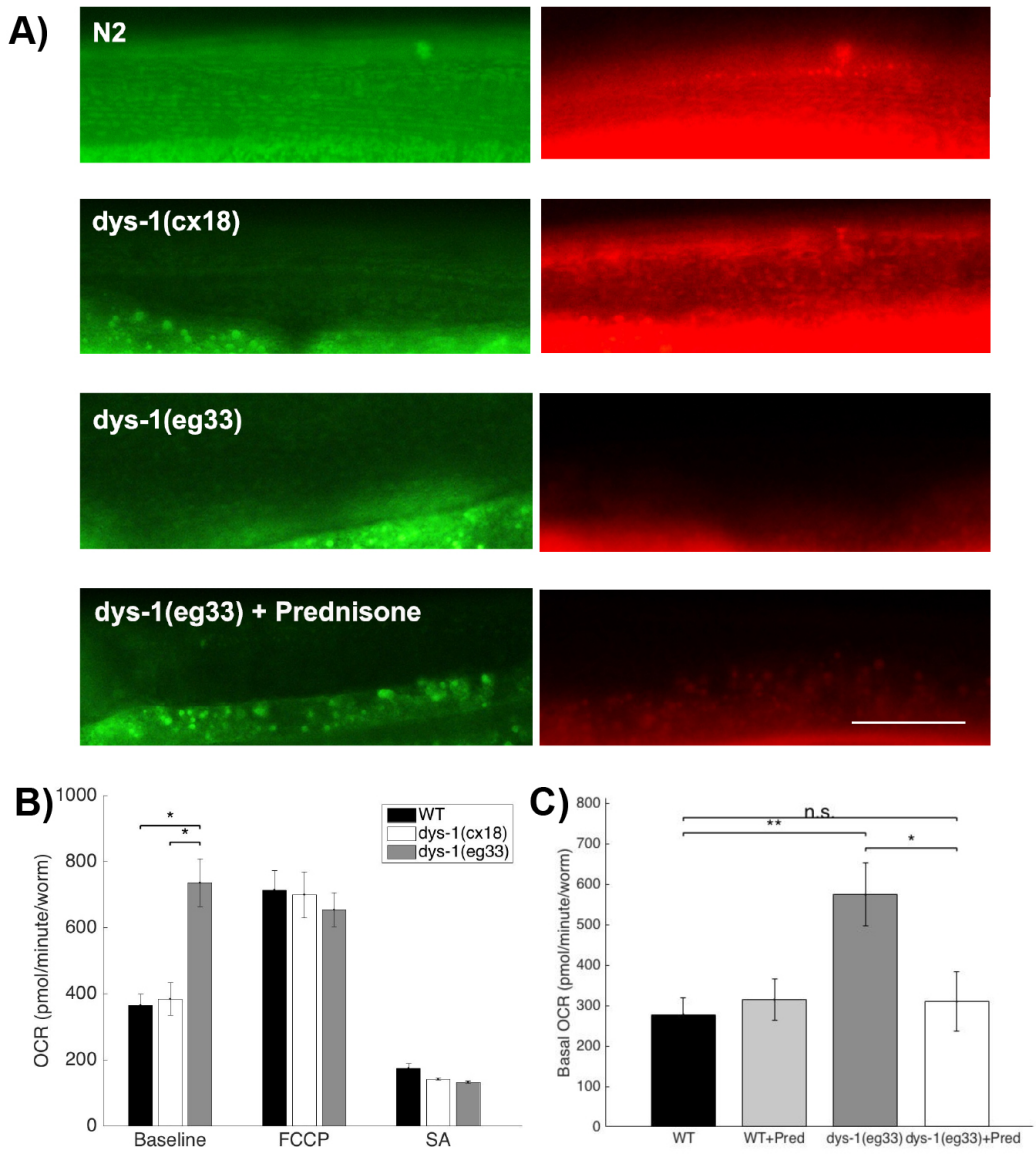
**Mitochondrial dysfunction is also a phenotype of *dys-1(eg33)*.** (A) JC-10- and MitoTracker Red-stained mitochondria show moderate depolarization of the mitochondrial membrane in *dys-1(cx18)* and severe depolarization in d*ys-1(eg33)*. This defect is not remedied by treatment with prednisone in *dys-1(eg33)*. Scale bar: 30 µm. (B) Compared with wild type (WT) and *dys-1(cx18)*, *dys-1(eg33)* has an abnormally high basal oxygen consumption rate (OCR), while maximal respiratory capacity is unaffected. Significances were assessed using a one-way ANOVA and Bonferroni multiple corrections. (C) Treatment with prednisone restores basal OCR to WT levels in *dys-1(eg33)* animals. Significance was assessed using a one-way ANOVA with Tukey's multiple comparison test. All OCR data are based on 20 worms per well with five wells per strain/condition. *P<0.05, **P<0.01; n.s., nonsignificant.

Having identified that mitochondrial network structure appears disrupted in *dys-1* mutants and that this is improved with prednisone treatment, we were curious whether mitochondrial function was similarly affected. We first used mitochondrial dyes to assess mitochondrial membrane potential. JC-10 is a dye that collects in the mitochondria based on membrane potential and exits as the mitochondrial membrane potential changes over time, as previously shown in another *C. elegans* mutant (Gaffney et al., 2015). Compared with wild-type animals, *dys-1(cx18)* has a somewhat reduced mitochondrial membrane potential, while *dys-1(eg33)* is more severely affected (Fig. 6A). To confirm these defects in mitochondrial membrane potential, we used a second dye, MitoTracker Red, which collects in the mitochondria based upon membrane potential, but, unlike JC-10, does not exit the mitochondria once inside (Gaffney et al., 2014). The MitoTracker Red accumulation matched that of JC-10 (Fig. 6A), demonstrating that the impaired membrane potential in the JC-10-dyed worms was not an artifact of loss of membrane potential during the staining procedure. Interestingly, with both dyes, prednisone does not improve the defect in membrane potential in *dys-1(eg33)*, indicating that improvements that we see in strength and thrashing rate in *dys-1(eg33)* under prednisone treatment can be attributed to a different mechanism.

In order to quantify the defect in mitochondrial function in *dys-1(eg33)* mutants, we assessed oxygen consumption rates (OCRs). Although *dys-1(cx18)* have normal OCR compared with wild type, *dys-1(eg33)* have abnormally high baseline respiration (Fig. 6B). Treatment with the uncoupling agent carbonyl cyanide-4-(trifluoromethoxy)phenylhydrazone (FCCP) revealed that unlike wild type and *dys-1(cx18)*, *dys-1(eg33)* had no statistically significantly detectable, spare respiratory capacity (Fig. 6B). No statistically significant differences in non-mitochondrial respiration, as assessed by treatment with sodium azide, were found between the strains. Thus, the lack of spare respiratory capacity in *dys-1(eg33)* is likely a key driver of the increased severity of muscle defects in *dys-1(eg33)* versus *dys-1(cx18)*.

Given that prednisone treatment improves muscle strength, thrashing rate and mitochondrial network integrity in *dys-1(eg33)*, we were interested in determining whether prednisone could also normalize the aberrantly high basal OCR. Indeed, treating *dys-1(eg33)* with prednisone returned basal OCR to wild-type levels (Fig. 6C). These results, combined with the lack of major effect of prednisone on mitochondrial membrane potential (Fig. 6A), suggest that prednisone has a predominant effect on mitochondrial respiratory function rather than restoring membrane potential. This is interesting, as Brouilly et al. (2015) recently showed that prednisone improves muscle membrane structure, including the mitochondria, in *dys-1; hlh-1*.

## DISCUSSION

### Strength as a novel phenotype for *C. elegans* DMD studies

In the present study, we demonstrate the ability to measure the strength of *C. elegans dys-1* mutants and detect functional improvements in muscle strength in *dys-1(eg33)* after treatment with compounds known to improve muscle health. Previously, there was not a means to directly measure the strength of *C. elegans*, but recently our group established a consistent and reliable strength measurement routine using our microfluidic NemaFlex device (Rahman et al., 2018). This has allowed us to demonstrate, for the first time, that strength deficiency is a phenotype of the *dys-1(eg33)* strain, which further represents *C. elegans* as a useful model for replicating some of the pathophysiologies of human diseases in nematodes.

For high-throughput drug screens with dystrophin-deficient *C. elegans*, it might not be feasible to measure a large quantity of parameters to quantify nematode health. We show here that the thrash assay detects deficiencies in both *dys-1* mutants and improvements under treatment with compounds. However, a decrease in thrashing rate does not necessarily correlate with a loss of muscle strength. For example, wild-type animals have lower thrashing rates on Days 3 and 5 than on Day 1, although there is not a strength decline at this same time point. Therefore, thrashing rate and muscle strength measures do not necessarily report on the same aspect of worm physiology. For the purpose of high-throughput drug screens, an automated version of the thrashing assay would be a quicker way of determining hits (Buckingham et al., 2014); we propose that our NemaFlex system would be useful in validating whether these drugs also improve the more clinically relevant measure of muscle strength. Further automation of our NemaFlex imaging and postimaging analysis protocol could help make NemaFlex more reasonable as a first-step screening assay; however, under the current protocol, throughput is somewhat limited and would thus be more appropriate as an assay to validate hits that come out of a thrashing-based drug screen or other high-throughput screening method.

Therefore, we propose that a direct measure of muscle function is perhaps the most valuable single measure to extract from drug screens. We recognize the value in assessing other physiological abilities, as *dys-1(eg33)* animals are also deficient in thrashing and burrowing. Advantages of our system over the previously reported burrowing assay (Beron et al., 2015) include the ability to culture the nematode over its whole life while maintaining individual worm identity and controlling the contents of the fluidic environment in a temporal manner. There is also no requirement of a stimulus for observation of the desired phenotype, which is easy to observe due to the transparency of the platform, which allows for clear imaging

Previous studies with dystrophin-deficient *C. elegans* have also looked at non-physiological measures that aim to assess the integrity of the muscle rather than the function. If muscle strength is improved under a certain drug treatment, previously described assays looking at non-physiological measures should then be used to further assess the efficacy of the treatment. Our transgenic *dys-1* strains expressing GFP in mitochondria of the body wall muscle that we report here are perfectly suited for this purpose, although other methods have previously been reported as well. Beron et al. (2015) looked at muscle degeneration in burrowing animals by tagging muscle cell nuclei and mitochondria with GFP, and others have looked at body wall muscle integrity after staining (Gieseler et al., 2000; Mariol and Ségalat, 2001). Looking at muscle cell integrity under a certain drug treatment could thus entail using the *dys-1; hlh-1* mutant or allowing worms to burrow in the presence of the drug of interest to hasten muscle damage in one of the single mutation *dys-1* strains.

### Differences in muscle strength and other phenotypes between *dys-1(eg33)* and *dys-1(cx18)*

The inability of NemaFlex to detect muscular defects in *dys-1(cx18)* in a crawling environment is not surprising, given that adult worms similarly aged to the ones studied here have no abnormal muscle cells and are indiscernible from wild-type animals based on this parameter (Gieseler et al., 2002). Additionally, the mitochondrial fragmentation is not as severe in *dys-1(cx18)* as in *dys-1(eg33)*. However, the question still remains on what the key differences are between *dys-1(eg33)* and *dys-1(cx18)* that lead to these drastic differences in muscle functionality, especially given that both animals are deficient in thrashing (our results here) and burrowing (Beron et al., 2015). Crawling, swimming and burrowing are kinematically distinct from one another and offer unique challenges for the worm; observing different phenotypes among these environments could result from this distinction. It is likely that the burrowing assay challenges the muscles in a way not done in NemaFlex. Burrowing relies on the head muscles, while the NemaFlex analysis selects for the maximum force exertion, typically coming from body wall muscles. Therefore, if head muscles were weaker, our system would not detect this under the current workflow. We also see that in the swimming worms, where both *dys-1(cx18)* and *dys-1(eg33)* are slower thrashers, *dys-1(cx18)* does not respond quite as strongly to the drug treatments.

Further assessment with these three unique functional readouts, along with future efforts targeting mechanistic questions, could help answer why *dys-1(eg33)* shows an impaired phenotype and *dys-1(cx18)* does not. Previous work with *C. elegans* has identified defects in calcium signaling and acetylcholine sensitivity as pathophysiologies associated with dystrophin deficiency, so it is possible that these defects are more severe in *dys-1(eg33)* than in *dys-1(cx18)* (Mariol and Ségalat, 2001; Zhan et al., 2014; Bessou et al., 1998; Giugia et al., 1999). However, both strains are also reported as having null mutations, indicating that neither strain should produce even a partially functional dystrophin product. It thus remains unclear why the worms exhibit some distinct phenotypes from one another, but our data reported in this paper support the notion that there are fundamental differences between *dys-1(eg33)* and *dys-1(cx18)*. The more severe phenotype of *dys-1(eg33)* in its levamisole resistance and basal OCR, as well as the temperature-sensitive nature of *dys-1(cx18)*, offer further perspective on why these strains differ from one another in their physiologies.

Oh and Kim (2013) previously showed that *dys-1(eg33)* has higher levels of GST-4 reporter than *dys-1(cx18)*. Increased *gst-4* expression leads to increased resistance to oxidative stress (Leiers et al., 2003), and this is entirely consistent with our OCR data for *dys-1(eg33)*. Additionally, we also showed that *dys-1(cx18)* display temperature sensitivity in their thrashing movement. Similar movement data for *dys-1(cx18)* were reported at 25°C (Hueston and Suprenant, 2009); thus, our data are consistent with published data. The nonsense mutation in *dys-1(cx18)* corresponds to termination at AA 2721, which is immediately before the start of spectrin repeat domain 5, which starts at AA 2725. The temperature-sensitive nature of the movement decline in *dys-1(cx18)*, but not *dys-1(eg33)*, suggests that *dys-1(cx18)* probably produces a partially functional protein in a temperature-sensitive fashion. This idea of more unfolding occurring at 25°C is consistent for other metastable temperature-sensitive mutations in *C. elegans* (Ben-Zvi et al., 2009).

### Prednisone and melatonin improve strength in *C. elegans*

The two pharmacological compounds that we test here, prednisone and melatonin, offer improvements in muscle strength and may also elucidate mechanisms behind muscle strength loss in muscular dystrophy. Previously, Gaud et al. (2004) reported that prednisone reduces the number of abnormal muscle cells in their *dys-1; hlh-1* model. We demonstrate here that prednisone gives a functional improvement in the *dys-1(eg33)* animal as well. Although *dys-1(eg33)* does not exhibit major defects in the sarcomeres like in the sensitized models, we can still detect and treat strength declines. This is in contrast to our past work with integrin attachment complex mutants, where both sarcomere and mitochondrial defects were present in animals that were detectably weaker (Etheridge et al., 2015). Our results here indicate that NemaFlex can detect alterations in strength in the absence of major structural defects in muscle, which raises the question of whether mitochondrial deficits, rather than very minor sarcomere deficits, underlie the detected loss of strength.

While we are able to detect functional improvements under both drug treatments, the exact mechanism by which prednisone helps to alleviate symptoms is not known, although efficacy is at least, in part, attributed to reduction of inflammation (Parrillo and Fauci, 1979; Mendell et al., 1989). Another proposed mechanism is protection against mechanically induced muscle damage (Jacobs et al., 1996). Also, little is known about the mechanism of melatonin in the treatment of dystrophin-deficient muscle, although it has been demonstrated to reduce oxidative stress markers in erythrocytes in blood samples from humans with DMD (Chahbouni et al., 2011). In DMD patients treated with melatonin, several measures scoring oxidation and inflammation were also improved over a 9-month treatment period (Chahbouni et al., 2010). Functional measures were not reported for this study, but *mdx* mice treated with melatonin show decreased creatine kinase levels and improved muscle function in another study (Hibaoui et al., 2011). These proposed mechanisms could be studied further using the *C. elegans* DMD model that we present here.

### *dys-1(eg33)* shows clinical relevancy

Given that *dys-1(eg33)* is weaker than the wild type and responds well to prednisone treatment, the standard treatment for muscular dystrophy in humans, we are convinced that this particular strain could currently be the most clinically relevant model of *C. elegans* for muscular dystrophy, especially when considering that much of the muscular dystrophy work has been done with the genetically sensitized strain, *dys-1(cx18); hlh-1*. Null mutations of *hlh-1*, although not inhibitory to muscle development, do lead to muscle that contracts poorly and animals that are uncoordinated (Chen et al., 1994). The *dys-1; hlh-1* mutant has been utilized as a way to strengthen the effects of the *dys-1* mutation on muscle degeneration (Gieseler et al., 2000).

While this sensitized worm may be useful for studying certain aspects of muscular dystrophy, its relevance to the mechanisms of muscular dystrophy in humans could be confounded by the presence of the additional mutation. As a result, any technique that offers a way to detect muscular defects or decreased function in muscle in worms with a mutation only in the *dys-1* gene arguably offers a large advantage over these previous assays. We propose that future work with *C. elegans* muscular dystrophy models should follow two main thrusts: screening novel compounds and probing mechanisms using *dys-1(eg33)*. Our platform is capable of identifying novel drugs or already approved drugs used for other purposes that improve muscle function in *dys-1(eg33)*. This could lead to clinical studies and may also help to unearth unknown mechanisms associated with dystrophin deficiency. Thus, answering mechanistic questions in future work is a huge priority.

### Conclusion

NemaFlex is a promising platform for screening compounds that could potentially help to alleviate the loss in muscle strength associated with muscular dystrophy. This allows us to study muscular dystrophy mechanisms and treatments in the worm without having to use sensitizing mutations. Subcellular analyses looking at mitochondrial integrity also enable further assessment of the health of muscle in *dys-1* mutants. The muscular weakness, thrashing deficiencies, mitochondrial fragmentation, impaired mitochondrial function and drug response of *dys-1(eg33)* indicate a clinically relevant model for future investigations in the worm. Determination of muscle strength, when paired with other previously established measures of worm physiology, muscle integrity and overall health, will offer a more robust method for determining novel compounds for treating dystrophin-deficient worms.

## MATERIALS AND METHODS

### Nematode strains and culture

*C. elegans* strains used in this study were wild-type N2, provided by the Driscoll Laboratory (Department of Molecular Biology and Biochemistry, Rutgers University, Piscataway, NJ, USA), and *dys-1(eg33)* (strain BZ33) and *dys-1(cx18)* (strain LS292), provided by the Caenorhabditis Genetics Center (CGC). Both mutants have nonsense mutations in the *dys-1* gene (Oh and Kim, 2013). We also used four new strains – CC96 [*dys-1(eg33) I; (jls01 (myo-3::GFP, rol-6 (su1006)); unc-54::lacZ V)*], CC97 [*dys-1(cx18) I; (jls01 (myo-3::GFP, rol-6 (su1006)); unc-54::lacZ V)*], CC90 [*dys-1(cx18) I; ccIs4251 I; him-8(e1489) IV*] and CC91 [*dys-1(eg33) I; ccIs4251 I; him-8(e1489) IV*] – generated for this study to evaluate sarcomere and mitochondrial network integrity in *dys-1(eg33)* and *dys-1(cx18)*, along with PJ727 [*jls01 (myo-3::GFP, rol-6 (su1006)); unc-54::lacZ V*] and CB5600 [*ccIs4251 (Pmyo-3::Ngfp-lacZ; Pmyo-3::Mtgfp) I; him-8 (e1489) IV*], also provided by the CGC. The PD55 strain was used for OCR experiments. Animals were maintained at 20°C (unless otherwise noted) on nematode growth medium (NGM) plates with *E. coli* OP50 using standard protocol. Animals for the study were age synchronized by transferring ∼30 gravid adult nematodes of each strain to the various agar plates (with or without pharmacological treatments) and then leaving them to lay eggs for ∼3 h. Adult animals were then removed, and the agar plates with eggs were left in the 20°C incubator for 3 days. Animal age is given as day of adulthood.

### Pharmacological treatments

**Fig. 7.**
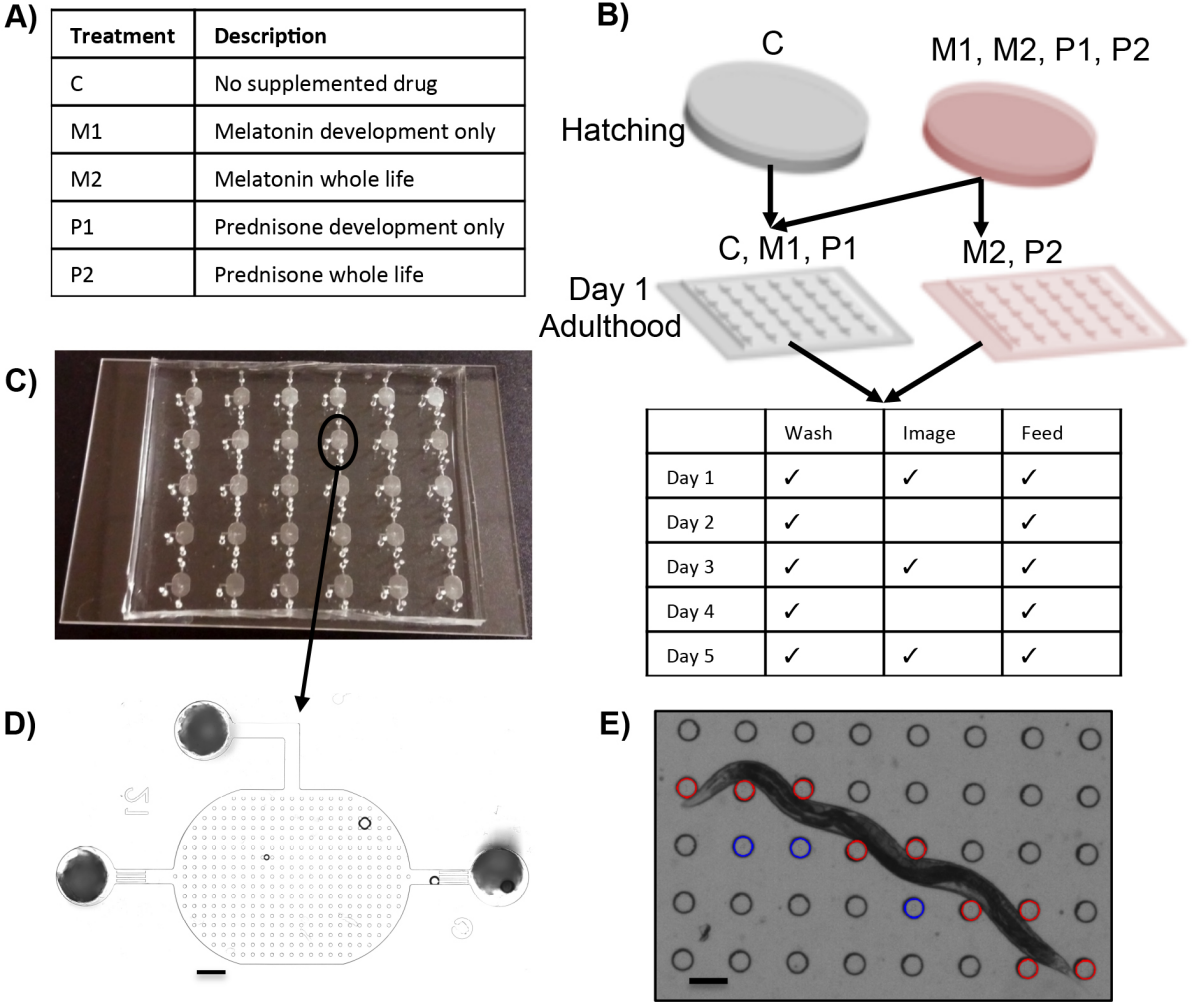
**Experimental protocol for testing the efficacy of pharmacological compounds and the microfluidic platform used from the beginning of adulthood.** (A) A summary of the different treatments and associated abbreviation used to describe each treatment. (B) Animals start out on agar for the first 3 days, when development is occurring, and all animals except the control group of each strain receive a pharmacological treatment (pink). On the first day of adulthood, all animals are transferred to the devices in which they are fed and imaged over the next few days; animals receiving lifelong treatment continue to receive compounds in the microfluidic device (shown in pink). (C) A view of the 30-chamber microfluidic chip used to house the nematodes from Days 1-5 of adulthood. The device is bonded on a standard 75×50 mm glass slide. (D) An image of a microfluidic chamber used to house a single worm. The deflectable pillars enable force measurement. Scale bar: 300 μm. (E) A close-up view of some of the pillars being tracked for deflection via the NemaFlex image-processing software. Pillars currently in contact with the worm are shown in red; pillars that are deflected in a different frame of the image sequence are shown in blue. Scale bar: 100 μm.

There were five different groups in this experiment for each of the three strains studied: no pharmacological intervention (control), melatonin or prednisone received during development only, and melatonin or prednisone received during both development and adulthood (Fig. 7A). NGM plates were prepared normally for the control groups. For the treatments, melatonin (Sigma-Aldrich) and prednisone (Sigma-Aldrich) were added to the NGM immediately after autoclaving to final concentrations of 1 mM and 0.37 mM, respectively. The prednisone concentration was chosen as 0.37 mM, as this is a concentration falling within the range of concentrations previously reported by Gaud et al. (2004) to reduce the number of damaged muscle cells in the *dys-1; hlh-1* model. Similarly, a concentration of 1 mM of melatonin is within the range of melatonin concentrations previously reported to affect physiology, specifically the number of body bends, in wild-type *C. elegans* (Tanaka et al., 2007). Thus, drug concentrations that were selected are values known to fall within the range of concentrations that affect animal physiology and/or muscle health. Animals that continued to receive treatment after development, corresponding to introduction to the microfluidic device on Day 1 of adulthood, received treatments at concentrations of 0.1 mM and 0.037 mM for melatonin and prednisone, respectively. Lower concentrations were used due to the more direct contact with the drug in the microfluidic device compared with the agar plates.

### Animal culture and imaging in microfluidic devices

When animals were ∼72 h posthatching, they were loaded into the microfluidic devices along with a solution of 100 mg of *E. coli* ml^−1^ of liquid NGM (NGM without the agar). For animals continuing to receive the pharmacological treatment after development, the compound was introduced into the *E. coli* solution at the appropriate concentration before the concentrated bacteria solution was added to the device (Fig. 7B). On each day for the remainder of the experiment, the devices were washed using liquid NGM to remove progeny and debris, and a fresh solution of bacteria was added to the device (Fig. 7C,D). The arena of pillars and barriers in the outlet ports allow for the retention of adult animals and the filtering out of unwanted progeny, as has been previously demonstrated for *C. elegans* maintenance in microfluidic devices (Hulme et al., 2010; Wen et al., 2012; Xian et al., 2013; Wen et al., 2014).

After clearing the devices of progeny and debris, and before adding fresh *E. coli*, animals were imaged in the microfluidic chambers (Fig. 7C,D) for 45-s episodes at a rate of five frames per second. A Nikon Eclipse TI-E microscope with Andor Zyla sCMOS 5.5 camera was used. Any animals that remained stationary during the first image sequence, although few in number, were re-imaged until a movie including sufficient worm locomotion was obtained.

### Strength measurements using NemaFlex

Deflections and strength measurements were obtained using standard NemaFlex processing protocol, which involves automated tracking of the deflectable pillars (Fig. 7E) (Ghanbari et al., 2012; Johari et al., 2013; Khare et al., 2015; Qiu et al., 2015). Pillar deflection values extracted during image processing were converted to forces using Timoshenko beam deflection theory (Etheridge et al., 2015; Rahman et al., 2018). We then obtained animal strength from these forces by selecting for the maximal force exerted in each frame of the acquired image sequence and selecting for the 95th percentile value (defined as *f*_95_) among these maximal forces. The *f*_95_ value for an individual worm is analogous to the maximum voluntary force in humans, and thus defines a measure of animal muscular strength. Further details on the methodology and data analysis can be found in Rahman et al. (2018), and the custom-built software can be obtained by directly contacting our laboratory. Animal strengths were compared using a two-sample *t*-test (MATLAB, R2015b), with each individual animal strength value being treated as an independent sample. The only animals excluded from the analysis were those for which the custom-built MATLAB software failed to process the movie, which can result from too many air bubbles inside the microfluidic devices or non-uniform illumination. Animal diameters were measured using ImageJ (https://imagej.nih.gov/ij/).

### Thrashing assay

To crosscheck whether worms lacking in strength also exhibit functional deficiencies in swimming, we used a simple thrashing assay (Gaffney et al., 2014). There were three different groups for each of the three strains studied [wild type, *dys-1(eg33)* and *dys-1(cx18)*]: no pharmacological intervention (control) and melatonin (1 mM) or prednisone (0.37 mM) treatment through the last day of assessment. Animals were age synchronized as described in the strength assay and maintained on NGM agar plates throughout the experiment. Animals were manually picked to new plates every other day during the egg-laying period.

On Days 1, 3 and 5 of adulthood, movement rates of the worms were recorded using a thrashing assay (also referred to as swim test). Thrashing assays were carried out by picking a worm into 20 µl M9 buffer on a microscope slide. The number of bends in 10 s was counted and repeated five times for each worm for three independent biological replicates. One body bend was recorded as one rightward body bend and leftward body bend. For each treatment, movement rates for ten worms were measured. The differences in movement rates between treatment groups were analyzed using a two-sample *t*-test in MATLAB. The same method was utilized for temperature sensitivity experiments, with the exception that animals were cultured at 25°C instead of 20°C and significance was assessed using a two-way ANOVA with Tukey's multiple comparison test.

### Levamisole sensitivity assay

To check for differences in levamisole sensitivity among wild type, *dys-1(cx18)* and *dys-1(eg33)*, the groups were exposed to levamisole hydrochloride (Sigma-Aldrich, 31742) at 100 µM in M9 buffer. Animals were placed in 2.5 ml levamisole in 30 mm Petri dishes. Starting from *t*=0 min, the numbers of paralyzed animals were scored every 10 min until all wild-type worms were paralyzed. Experiments were performed for populations of Day 1 adult worms cultured at 20°C or 25°C. For worms cultured at 20°C, two independent biological replicates were performed, where *n*=50 for each experiment (total *n*=100 per strain). For 25°C, a single experiment was performed where *n*=50 per strain.

### Sarcomere structure

To determine whether *dys-1(cx18)* and *dys-1(eg33)* worms showed defects in sarcomere structure, the worms were stained with Rhodamine Phalloidin Stain (Invitrogen, R415). The phalloidin staining procedure was carried out as described by Gieseler et al. (2000).

In addition to actin staining using phalloidin, crosses were made using PJ727 [*jls01 (myo-3::GFP, rol-6 (su1006)); unc-54::lacZ V*], which has GFP fusion proteins localized to the contractile apparatus, with *dys-1(eg33)* and *dys-1(cx18)*. The resulting crosses were referred to as CC96 [*dys-1(eg33) I; (jls01 (myo-3::GFP, rol-6 (su1006)); unc-54::lacZ V)*] and CC97 [*dys-1(cx18) I; (jls01 (myo-3::GFP, rol-6 (su1006)); unc-54::lacZ V)*]. Images were taken on Days 0, 1, 2 and 3 of adulthood. All images were taken at 40× magnification using a Nikon Eclipse 50i microscope.

### Mitochondrial strains and imaging

The CB5600 [*ccIs4251 (Pmyo-3::Ngfp-lacZ; Pmyo-3::Mtgfp) I; him-8 (e1489) IV*] strain, which has GFP fusion proteins localized to muscle mitochondria and nuclei, was used for this study. Crosses were made between the CB5600 strain and *dys-1(cx18)* (LS292 strain) and *dys-1(eg33)* (BZ33 strain). The resulting strains were CC90 [*dys-1(cx18) I; ccIs4251 I; him-8(e1489) IV*] and CC91 [*dys-1(eg33) I; ccIs4251 I; him-8(e1489) IV*]. CB5600 was used for the wild-type imaging. On Days 1, 3 and 5 of adulthood, animals were imaged in 20 µl M9 buffer on a microscope slide with a cover slip. All images were taken at 40× magnification using a Nikon Eclipse 50i microscope.

### OCR

To investigate DMD-mediated changes in mitochondrial function, OCR measurements were performed using the Seahorse XFe24 analyzer (Agilent), in line with previously described methods (Koopman et al., 2016). On Day 0 of adulthood, wild-type, *dys-1(cx18)* (LS292 strain) and *dys-1(eg33)* (BZ33 strain) animals were washed twice in M9 buffer and transferred into M9-filled wells (20 worms/well) in replicates of five per condition (i.e. five wells per strain). To generate stable OCR measurements, five measurement cycles were performed for basal OCR, nine cycles for maximal OCR following the addition of FCCP (10 µM final well concentration) and five cycles for non-mitochondrial OCR, following the addition of sodium azide (40 nM final well concentration). A follow-up experiment was conducted to investigate whether prednisone treatment could rescue DMD-mediated changes in basal OCR. To do this, basal OCR was measured, as described, in adult (Day 1) wild-type (N2) and *dys-1(eg33)* animals both with and without prednisone treatment (20 worms/well, five replicates). Prednisone-treated worms were cultured, as previously described, on prednisone-treated (0.37 mM) agar. OCR measurements were normalized to the number of worms per well. To avoid unstable OCR measurements, the final three, seven and two measurement cycles were used for the statistical analysis of basal, maximal and non-mitochondrial OCR, respectively. Differences in OCR were detected with a one-way ANOVA with Tukey's multiple comparison test using GraphPad Prism 6. The α-level of significance was set at *P*<0.05.

### JC-10 and MitoTracker Red staining

To assess mitochondrial membrane potential, two *in vivo* dyes, JC-10 (Enzo Life Sciences, 52305) and MitoTracker Red CMXRos (Invitrogen, M7512), were used. Strains used for measuring mitochondrial membrane potential were wild type (N2), *dys-1(cx18)* (LS292 strain) and *dys-1(eg33)* (BZ33 strain). For prednisone-treated worms, animals were cultured as previously described on agar containing prednisone at a concentration of 0.37 mM. On the first day of adulthood, 40 worms were picked into 83 µM JC-10 in freeze-dried OP50 solution (LabTIE) for 4 h before imaging. The worms stained with MitoTracker Red were imaged on the first day of adulthood and the protocol by Gaffney et al. (2014) was followed. Representative images are shown for each strain stained with JC-10 and MitoTracker Red.
